# The health related quality of life of people living with HIV/AIDS in sub-Saharan Africa - a literature review and focus group study

**DOI:** 10.1186/1478-7547-8-5

**Published:** 2010-04-16

**Authors:** Bjarne Robberstad, Jan Abel Olsen

**Affiliations:** 1 Research Group Global Health: Ethics, economics and culture, Centre for International Health and Department of Public Health, University of Bergen, Norway; 2 Department of Community Medicine, University of Tromsø, Norway

## Abstract

**Background:**

While health outcomes of HIV/AIDS treatments in terms of increased *longevity *has been the subject of much research, there appears to be very limited research on the improved *health related quality of life *(HRQL) that can be applied in cost-utility analyses in Africa south of the Sahara (SSA). Most of the literature that does exist present HRQL measured by disease specific instruments, but such data is of little use as input to economic evaluations.

**Methods:**

A systematic review of the literature on HRQL weights for people living with HIV/AIDS in Africa was performed, and the findings are presented and interpreted. We also use focus group discussions in panels of clinical AIDS experts to test the preference based on a generic descriptive system EQ-5D. We contrast quality of life with and without antiretroviral treatment (ART), and with and without treatment failure.

**Results:**

In only four papers were the HRQL weights for HIV/AIDS in sub-Saharan Africa estimated with generic preference based methodologies that can be directly applied in economic evaluation. A total of eight studies were based on generic health profiles. While such 'health profiles' are not preference based, the scores could potentially be transformed into health state utilities. Most of the available literature (20 papers) utilized disease specific instrument, which are not applicable for economic evaluation.

The focus group discussions revealed that HRQL weights are strongly correlated to disease stage. Furthermore, clinical experts consistently report that ART has a strong positive impact on the HRQL of patients, although this effect appears to rebound in cases of drug resistance.

**Conclusions:**

EQ-5D appears to be an appropriate tool for measuring and valuing HRQL of HIV/AIDS in Africa. More empirical research is needed on various methodological aspects in order to obtain valid and reliable HRQL weights in economic evaluations of HIV/AIDS prevention and treatment interventions.

## Background

There is high international pressure to allocate more resources on treatment programmes for people living with HIV/AIDS [1], which in it's own respect is important given the magnitude of the impact of the epidemic. A crucial issue in the evaluation of alternative programmes across different disease groups is the comparison of health outcomes with costs. Many studies have focused on health outcomes in terms of increased longevity from HIV/AIDS treatments [2-5], but there appears to be very limited research measuring outcomes in terms of improved health related quality of life (HRQL) or improved disability weights (DW).

Economic evaluations of HIV/AIDS interventions can largely be divided into two groups; studies that ignore improvement in health related quality of life and those that seek to capture such improvements. While the former group of studies focus on simple clinical outcomes such as mortality or averted cases of HIV, the latter attempt to capture effect changes both in terms of life expectancy and improvements in health states. This is typically being done either by estimating quality adjusted life year (QALY) or disability adjusted life year (DALY). A basic premise for QALY analyses is that they depend on HRQL weights that should reflect peoples preferences [6]. Cost per DALY analyses depend on disability weights, that in country specific applications also are meant to be adapted to local circumstances [7].

Patients with HIV/AIDS are routinely categorized into one of four clinical disease stages of the WHO staging system (CS I - IV). The clinical stages involve different degrees of impaired health related to different dimensions of health that are affected, e.g. anxiety, pain and functioning. Given that medications may improve the different dimensions of health to varying extents, it becomes important to compare the improvements on a commensurable scale. Suitable methodologies for cost-utility analysis (CUA) would mean using generic descriptive instruments or a generic health scale [8]. Furthermore, since people's experiences of impaired health and their preferences for health reflect norms and cultural settings, the health state utility weights should ideally be obtained from a similar cultural setting.

Tengs and colleagues performed a meta analysis of utility estimates for HIV/AIDS. Based on studies from high income settings, they calculated pooled utility weights of 0.70 for AIDS, 0.82 for symptomatic HIV and 0.94 for asymptomatic HIV [9]. Our literature review revealed only three studies in sub-Saharan African (SSA) settings that explored the health related quality of life for people living with HIV/AIDS using methods that are appropriate for CUA [10-13]. While these studies represent important contributions, especially given the paucity of data in the area, they are fairly narrow in terms of methodological approaches and geographical setting. Two of the studies are from South Africa, while one study was undertaken in a Ugandan population.

This lack of studies from SSA is a paradox as roughly two-thirds of all people with HIV/AIDS are living and dying in this region [14]. Hence, it appears that many of the economic evaluations of HIV/AIDS interventions targeting SSA use quality of life or disability weightings which are largely unsupported by relevant evidence. In this paper, we summarize the available evidence on health related quality of life in people living with HIV/AIDS. We also present the results from nominal group discussions between clinical experts with experience from Ethiopia and Tanzania. This is a starting point for planned investigations at district hospitals in which patient preferences will also be elicited.

The main objectives of this paper are twofold. First, we present a review of the existing evidence on health related quality of life in HIV/AIDS patients in sub-Saharan Africa and consider how this information is used in the economic evaluation literature. The larger body of research using instruments that are not directly applicable in economic evaluation, will also be reviewed, though in less detail. Given the limited availability of studies that have been based on preferences for HRQL as experienced in the specific disease stages, and in the relevant cultural settings, our second objective reflects a recent research initiative: To test the appropriateness of an instrument designed to estimate HRQL in all four stages of HIV/AIDS. In this instrument, a direct Visual Analogue Scale (VAS) approach and an indirect descriptive system (EQ-5D) is applied with a panel of clinical AIDS experts. This instrument specifically seeks to contribute to better quality of life information on: patients in the different disease stages; patients who do or do not receive antiretroviral treatment (ART), and; patients who are or are not experiencing treatment failure.

Estimating HRQL-weights for disease is important for several reasons, such as monitoring the health status of individual patients and establishing levels of health for patient groups [15]. Perhaps the most common application of HRQL weights is in the calculation of quality adjusted life years (QALYs) for use in economic evaluations. The latter reason raises two important issues: i) what evidence is available for HRQL on HIV/AIDS in SSA, and; ii) what evidence has been used in weighting QALYs in the economic evaluation literature? The same issues are also considered for disability adjusted life years (DALYs), which are conceptually related to QALYs. Economic evaluation is important for priority setting in low income countries, where resources are so constrained that neither prevention nor treatment are being carried out at sufficient levels [16]. QALYs and DALYs have the same policy purpose of aiding priority setting decisions across disease areas. While both metrics are concerned with measuring qualitatively different types of health gains in a *commensurable *- or generic - unit, only QALYs claim to be preference based.

## Literature review

### Methods

**Figure 1:**
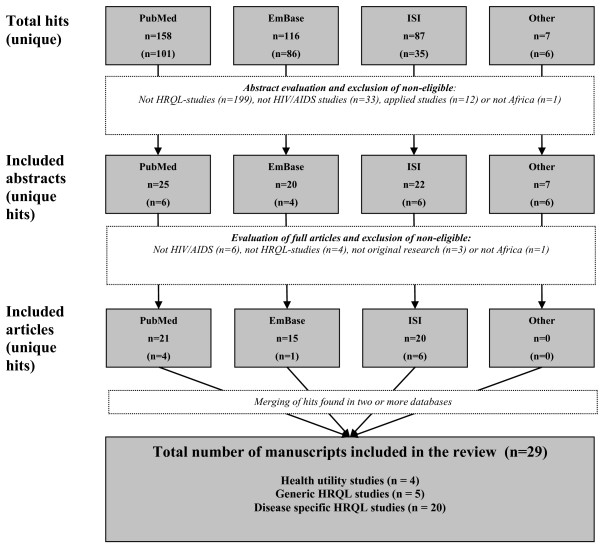
**Overview of the literature search, inclusion and exclusion procedures**.

We searched for literature in the databases PubMed, EmBase and ISI using the key words "HIV OR AIDS", "Africa south of the Sahara" AND "health related quality of life". A few abstracts based on expert input were also included. The total number of different hits of this process was 288. Detailed search strategies varied slightly with the different databases and are available from authors upon request together with the complete list of hits. The abstracts were screened for eligibility, using the following exclusion criteria: studies that clearly did not present HRQL data (n = 199); studies which were not about HIV/AIDS (n = 33); applied economic evaluations that were not primary sources of HRQL data (n = 12), and; studies not related to sub-Saharan Africa (n = 1). For the remaining abstracts (n = 43), the full articles were obtained and evaluated. After full article evaluation we excluded studies that turned out not to present data on HIV/AIDS (n = 6), did not present HRQL data (n = 4), that were not original research articles (n = 3) or turned out not to address sub-Saharan Africa (n = 1). A detailed description of this process is presented in Figure 1.

The remaining 29 research articles contain various types of health related quality of life evidence for people living with HIV/AIDS in sub-Saharan Africa. The majority of these studies (n = 20) assess quality of life using various *disease specific *descriptive systems. Such instruments may provide important information for clinical considerations and for monitoring treatment and development of individual patients. For economic evaluation, however, the outcome units used are of limited value because they are *incommensurable *across disease areas, and incommensurable with the valuation of the duration of the quality improvement, i.e. quality and quantity of life cannot be measured on the same metric. Eight of the studies assessed HRQL using generic instruments and health profiles, and several of these instruments can potentially be combined with value sets to present health state utilities. Preference based HRQL weights - or utility estimations - were not done for five of these studies, and the evidence is therefore not directly applicable in economic evaluations. Only four papers present preference based HRQL weights that can be directly applied in economic evaluations. Brief summaries of this evidence are provided below.

### Results

#### Quality of life utility estimates

The papers by Hughes et al. [10] and Jelsma et al. [11] reports HRQL in AIDS patients from a primary health care setting in Khayelitsha, South Africa. Hughes and colleagues found that health related quality of life is severely compromised in stage III and IV patients [10]. They used the EQ-5D descriptive system to compare quality of life of subjects from the general population with HIV patients not yet receiving ART. A main finding was that people with HIV had significantly more limitations across all the five health dimensions of the EQ-5D instrument (mobility; self care; usual activities; pain/discomfort; anxiety/depression). They also found mean scores on a [0-100] visual analogue scale (VAS) of 60.4 for people with HIV, compared to 80.1 for the general population. An overview of the studies presenting HRQL utility estimates is given in Table 1.

**Table 1:** Overview of studies presenting preference based HRQL weights for HIV/AIDS in sub-Saharan Africa.

Author	Setting	AIDS population	Sub-analyses	VAS^1)^	TTO	SG	EQ-5D index
Hughes	South Africa	WHO stages 3 or 4	General community	0.80			
(2004)	(Cape Town)	or CD4<200	Awaiting ART	0.60			

Jelsma	South Africa	WHO stages 3 or 4	General community	0.80			
(2005)	(Cape Town)	or CD4<200	Awaiting ART	0.62			
			ART (1 month)	0.70			
			ART (3 months)	0.71			
			ART (6 months)	0.74			
			ART (12 months)	0.76			

Louwagie	South Africa	WHO stage 4 or	ART (own health)	0.66			0.80
(2007)	(Free State)	CD4<200	Awaiting ART	0.62			0.69

Lara	Uganda	WHO stages	Own health (ART and non ART)	0.50 and 0.55			
(2008)	(Entebbe)	2, 3 or 4	WHO stage 2 (ART and non ART)	0.59 and 0.63	0.75 and 0.78	0.50 and 0.51	
			WHO stage 3 (ART and non ART)	0.39 and 0.39	0.49 and 0.52	0.34 and 0.39	
			WHO stage 4 (ART and non ART)	0.17 and 0.15	0.20 and 0.27	0.19 and 0.19	
			Own health reassessed (ART and non ART)	0.78 and 0.66			

1) VAS scores transformed from 0-100 scale to 0-1 scale to improve comparability.

While Hughes focused on baseline quality of life, the effect of antiretroviral treatment (ART) in the same patient population is reported by Jelsma and colleagues [11]. Their main conclusion is that health improvements from ART are good for all the five EQ-5D health dimensions, even in resource poor settings. The mean VAS scores progressively improved from 61.7 at baseline to 76.1 after 12 months on treatment with a triple therapy, but most of the improvement occurred as early as one month after start of treatment [11]. Neither of the two studies used the instruments to produce EQ-5D indices by applying population specific value sets. In other words, these studies did not infer corresponding HRQL weights from the EQ-5D combinations that patients had stated. Instead, the HRQL weights used in these studies were based on the EQ-VAS scores only. While VAS is a non-choice methodology that is considered theoretically inferior to the choice based time-trade-off methodology on which most EQ-5D tariffs are based, the EQ-VAS instrument is included as part of the EQ-5D procedure for measuring health http://www.euroqol.org.

In a large study from Free State province in South Africa, Louwagie estimate HRQL in a wide-scale roll out of ART in South Africa [13]. Like the Khayelitsha studies, the EQ-5D framework is used in the assessment. They found that patients waiting to start ART treatment commonly reported health problems. The two most commonly mentioned dimensions were pain/discomfort (57%) and depression/anxiety (42%). The mean EQ-VAS score for patients awaiting treatment was 62, which improved considerably for patients on treatment at 70 [13]. This supports the conclusion of Jelsma et al. [11] that ART is effective in improving people's self reported HRQL. Unlike the Khayelitsha studies, Louwagie and colleagues also converted the EQ-5D profiles for each patient into a single weighted EQ-5D index. This resulted in mean HRQL weights of 0.69 for patients awaiting ART, while the weight for those on ART was significantly better at 0.80 [13]. The basis for these weights was the standard UK tariff [17], which may not reflect the preferences for health in this South African population.

The fourth and most recent publication presenting HRQL weights of AIDS in SSA is from a Ugandan setting. This study by Lara and colleagues is the only evidence available from outside South Africa that is appropriate for application in economic evaluation of ART in SSA. While the South African studies present estimates for the patients' real-time perceived health, the Lara study in addition ask people living with HIV/AIDS about their preferences for a set of predefined health states representing WHO clinical stages 2, 3 and 4, respectively [12]. In this way they manage to assess utilities of a wider range of health states than those captured by the South African studies. They do this by applying VAS, time trade off (TTO) and standard gamble (SG) techniques, but they are not utilizing the EQ-5D or any other multidimensional generic descriptive system. The VAS is a metric representing the relative standing of health states on a "thermometer" ranging from "worst imaginable" to "best imaginable" health. The TTO and SG, on the other hand, imply trade-offs between life years and risks of good and bad health outcomes, respectively.

The VAS scores for people waiting to start ART treatment is very similar in the three South African studies, ranging from 0.60 to 0.62 [10,11,13]. In the Ugandan study, the participants were allowed to reassess their own VAS scores after having responded to the TTO and SG questions for the predefined health states. In the reassessment they considered their own health to be better than in the initial valuation. For the patients waiting to start treatment, the scores increased from 0.55 to 0.66 after reassessment [12].

While the studies referred to above present preference based HRQL weights on a [0-1] scale that enables *quality *of life to be measured in the same metric as *quantity *of life, the next class of studies present so-called 'health profiles'. These profiles represent generic measures of health, but they are not preference based, and, furthermore, the HRQL scores are *incommensurable *with quantity of life.

#### Generic HRQL profiles

A total of eight studies apply generic HRQL profiles, three of which had also used utility estimates (the South African papers referred to above). An overview of this literature is given in Table 2. Two of the studies were done before widespread introduction of antiretroviral treatment in Africa. O'Keefe and Wood compared the quality of life in people with HIV/AIDS in Western Cape, South Africa with a sample from the general population using the SF-36 instrument [18]. The main finding was that HIV subjects scored significantly lower than the controls on all eight health dimensions included in the SF-36. Furthermore, it was found that most of this decline in function occurred early in the disease (WHO stages I and II) [18]. A limitation of the study is that people with advanced AIDS were excluded, which prevents quantifying the full value of HIV-preventive interventions. Sebit and colleagues used the WHOQOL instrument to compare traditional medicine with conventional medical care. They found that WHOQOL is an appropriate measure of quality of life in people living with AIDS, and that traditional medicine has a role in improving quality of life [19]. It may be mentioned, however, that this is an observational study, and that selection procedures differed between the two treatment arms. Moreover, since the so-called conventional medical care does not include the use of antiretroviral drugs, the continued relevance of these findings may be questioned.

**Table 2:** Overview of studies presenting HRQL values based on generic health profiles for HIV/AIDS in sub-Saharan Africa.

1st Author	Setting	AIDS population	QoL measure	Authors' main conclusions
O'Keefe (1996)	South Africa (Western Cape)	WHO stages 1-4. Outpatients.	SF-36	HIV subjects scored significantly lower on all sub-scales compared to controls. The decline in function occurred early in disease by WHO stages 1 and 2. Insignificant differences in functioning between different CD4 strata.

Sebit (2000)	Zimbabwe (Harare)	Various stages, excluding the most severely ill.	WHOQOL	WHOQOL is a good measure of quality of life for patients with HIV infection. Phytotherapy (traditional medicine) has a role in improving QoL.

Kaaya (2002)	Tanzania (Dar es Salaam)	HIV positive women attending antenatal clinics	SF-36 and HS CL-25	Good correlation between SF-36 scores and HSCL-25. HSCL-25 is useful for screening of depression, but not sufficiently informative to gauge severity and inform management of depressive disorders.

Hughes (2004)	South Africa (Cape Town)	WHO stages 3-4, or CD4<200. Receiving HAART.	EQ-5D VAS + profiles	HRQL is severely compromised in stages 3 and 4, including the four EQ-5D domains of mobility, usual activities, pain/discomfort and anxiety/depression. The domain self care less affected.

Jelsma (2005)	South Africa (Cape Town)	WHO stages 3-4, or CD4<200. Receiving HAART.	EQ-5D VAS + profiles	Even in resource poor settings HRQL can be greatly improved by treatment with HAART, and there seems to be negligible impact from side-effects of the drugs. Improvements were found for all the five EQ-5D dimensions of health, but largest for pain/discomfort.

Nuwagaba-Biribonwoha (2006)	Uganda (Kampala)	HIV positive and negative women attending antenatal care.	Dartmouth COOP	Dartmouth COOP was found to be acceptable and feasible, and showed that HIV adversely affects maternal QoL among pregnant women. HIV positive women had poorer scores on six out of nine health dimensions.

Louwagie (2007)	South Africa (Free State)	WHO stage 4 or CD4<200. Receiving HAART.	EQ-5D VAS+index	EQ-5D was highly sensitive to HAART, with improvements after initiation of treatment on all five health dimensions. This supports its use in future evaluation of HIV/AIDS care. Results suggest that HAART if effective in improving people's self reported HRQL.

McInerney (2008)	South Africa (KwaZulu-Natal)	Patients > 18 years receiving HAART.	SF-36	Individuals who reported a greater length of time on medications, fewer co-morbid health problems, and greater social support had better physical functioning.

Two studies address the quality of life of pregnant HIV positive women. In a study from Tanzania, Kaaya et al. evaluate screening of depression in antenatal care by using the generic SF-36 instrument together with the disease specific Hopkins Symptoms Checklist-25 (HSCL-25) to [20]. The HSCL-25 was found to correlate well with the SF-36. In Uganda, Nuwagaba-Biribonwoha and colleagues found that HIV adversely affects maternal quality of life [21]. They used the Dartmouth COOP instrument in the assessment, and found it to be both acceptable and feasible. HIV positive women had poorer scores on six out of nine health dimensions in this study. The findings from these two studies cannot be compared, as the valuation instruments are not commensurable.

Several recent studies report quality of life profiles of patients with access to antiretroviral treatment. The quality of life findings from a clinical trial in Cape Town are reported in two publications. A main finding presented by Hughes and colleagues was that people with HIV had significantly more limitations across all the five health dimensions of the EQ-5D instrument [10], while Jelsma et al. found that antiretroviral treatment improved along all five dimensions, but especially for pain/discomfort [11]. The conclusion that ART is effective in improving people's self reported HRQL is shared also by one other South African study. In Free State, Louwagie et al. found clear evidence that ART effectively improves self reported HRQL [13]. Like Jelsma, they found improvement on all the five dimensions of EQ-5D, although the magnitude of the improvement was somewhat smaller [13]. Interestingly, all three studies find that the EQ-5D instrument is an appropriate tool for assessment of HRQL in AIDS in Africa [10,11,13].

The most recent publication included in this review is from KwaZulu-Natal, also in South Africa. McInerney and colleagues assessed how physical functioning for adults receiving ART is related to different medical and social variables. To assess HRQL they used the SF-36 instrument and found evidence that treatment duration, less co-morbidity, and better social support improved physical functioning [22].

While the above studies used *generic *descriptive systems to measure HRQL, the studies below have applied *disease specific *descriptive systems.

#### Disease specific HRQL evidence

The largest amount of HRQL evidence on HIV/AIDS is based on descriptive instruments that address issues of specific relevance to the disease, with social stigma as a typical example. Such disease specific information can be useful for clinical purposes but is not very useful for economic evaluation, because the measures of outcome are not comparable across disease and patient groups. Some of the instruments are not even comparable within the same disease.

The 20 studies included utilized a total of 18 different AIDS specific instruments, with MOS HIV being the most commonly applied (three publications). The WHOQOL BREF, WHOQOL HIV and HAT-QOL instruments were also used in at least two different studies each. The 20 different studies focused on a wide range of different areas, including various types of mental health, oral health and alternative medicine. This list of references is available from the authors upon request.

### Discussion

Our review of the economic evaluation literature on HIV/AIDS interventions in SSA confirms that the weights assigned to QALYs in most of the cases are more or less arbitrary. We argue that the DALY weights used in many influential economic evaluation studies on HIV/AIDS have an insufficient evidence basis as well. DALYs were used to calculate health effects in two influential review papers on the cost-effectiveness of HIV/AIDS interventions in Africa [23,24], as well as in many of the underlying original publications. The DALY is also the core methodology of a recent WHO-based publication [25]. It is potentially problematic when country specific studies fail to apply disability weights adapted to local circumstances, because it is then unknown whether the analyses reflect local values and consequently whether they will lead to priorities in concordance with population preferences.

The disability weights used in the DALY calculations are all taken from the Global Burden of Disease study, in which the values 0.123 and 0.505 are applied for HIV and AIDS, respectively [26]. Note that these values should be interpreted as 'inverse health scores' compared to QALY weights. When subtracted from 1.0, which is the state without disability in the QALY framework, the DALY weights roughly correspond to QALY weights [27]. A major difference is that DALY weights are standardised and based on expert views rather than the preferences of patients or population samples. Furthermore, the DALY weights for HIV/AIDS are very blunt in terms of clinical stage and disease progression, and do not distinguish between patients receiving or not receiving treatment. In the Hogan study, the issue of treatment was dealt with by making the assumption that people receiving ART have the same disability weight as people with HIV [25].

A couple of recent economic evaluations use QALYs as the outcome measure for ART. In a study addressing early versus late provision of ART in southern African adults, Bachman used the HRQL weights estimated in Khayelitsha by Hughes and colleagues [10,28]. Cleary et al. used the Khayelitsha data presented by Jelsma [11,29], and these were converted to utilities using a value set from the UK [17,29]. Although the UK value set is widely used, it is not necessarily relevant in settings that are culturally and economically completely different from Europe.

### Conclusions

This review reveals several knowledge gaps on health related quality of life for people with HIV/AIDS in SSA, and particularly so for evidence that can be applied in economic evaluations: i) no studies combine generic descriptive systems, such as the EQ-5D, with locally developed value sets; ii) except for one study [11] little is known on how patients' HRQL respond to ART treatment over time; iii) more knowledge is needed on the impact the perspective has (patient versus clinical expert) on the disability weights, since this may be needed to inform a revision of the DALY weights, and; iv) more knowledge on HRQL indices is needed for population groups in sub-Saharan Africa, especially outside South Africa.

## Focus group study

### Methods

We wanted to explore how clinical expert working in low income settings in sub-Saharan Africa judge the health related quality of life for different categories of people living with HIV/AIDS. Our study applied the EQ-5D questionnaire which consists of two parts; the generic descriptive system and the EQ-VAS (Visual Analogue Scale) [15]. Typically, the EQ-methodology is intended to be applied on patients, in order to allow individual preferences to be accounted for. However, expert opinions often represent important supplementary information, since experts would have better overview of the clinical picture than individual patients.

The EQ-5D system was chosen because it is simple to use, and has been demonstrated to produce valid results for a wide range of health conditions (http://www.euroqol.org provides more than 1,800 references). By applying an existing tariff, the EQ-5D model produce HRQL-indices for the various health combinations in the descriptive system. While the EQ-5D descriptive system is an indirect approach to eliciting health state preferences, the VAS directly asks the respondent to assign a quality of life score on a vertical 0-100 thermometer.

These two instruments were applied on the four severity levels of HIV/AIDS as defined by the WHO clinical staging system [30]. Roughly, stage I is non-symptomatic HIV positive status, stage II is mild, stage III is advanced, while stage IV is severe AIDS in its terminal phase. In addition, we distinguished between non-treated conditions and HIV/AIDS treated with antiretroviral treatment (ART) for stages II, III and IV. Finally, for stage IV we distinguished between ART with and without treatment failure, so the total number of different health states under consideration is eight. For all health states, it was stressed that the respondents should think about average patients at average points in time of progression through each stage.

We organized three nominal group discussions with 10 experts who have clinical experience from Ethiopia and Tanzania. First, the experts individually assessed the HRQL associated with each HIV/AIDS stage using the EQ-5D descriptive system as well as the EQ-VAS. They were then challenged to reach a consensus in a nominal group discussion with the authors as moderators. This is in contrast to the EQ-5D protocol, where disease weights should be elicited from patients. The existence of any difference between experts (experienced clinicians) and patients will be explored based on data from a newly initiated research project at a hospital in Tanzania.

The EQ-5D indices were calculated using the most commonly applied UK tariff [17] as well as a more recent Zimbabwe value set [31]. The UK tariff is based on a British household survey that used the time-trade-off (TTO) approach. However, peoples' experience and views on health may be quite different in a setting with different cultural and economic conditions such as those typically found in SSA. Therefore, we also applied the Zimbabwe tariff, which - like the UK-tariff - is based on the TTO-approach [31]. The key differences between the UK and the Zimbabwe tariffs are that the latter generally gives higher values, particularly to those states with reductions in i) mobility; iv) pain/discomfort, and; v) anxiety/depression. The exceptions are reduction in usual activities (both levels 2 and 3) and level 3 of self-care, which is considered to be more severe in Zimbabwe than in the UK. Differences in tariffs are likely to reflect more general differences between the two countries in people's life and health expectations.

### Results

**Figure 2:**
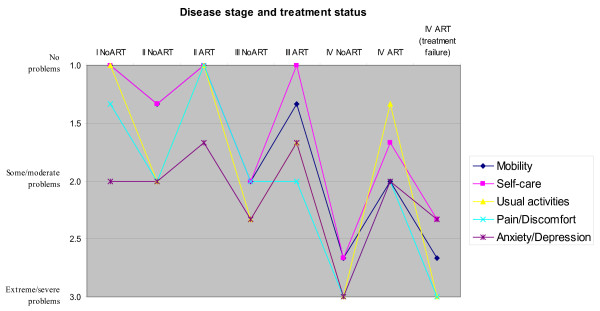
**Average level for each of the five health dimensions of EQ-5D depending on disease progression (Clinical stages I-IV) and whether patients receive antiretroviral treatment (ART) or not (No ART)**.

The participating experts had no problems responding to the EQ-5D exercise. The results of the nominal group discussions are illustrated in Figure 2. Generally, the experts consider anxiety and depression to be the dimensions that most severely affect HIV/AIDS patients. Naturally, health tends to worsen as the disease progresses, but the experts almost consistently judged the condition to improve considerably for all five dimensions with the introduction of ART. The major exception is in the case of treatment failure for stage IV patients, who were considered to be almost equally ill as stage IV patients without ART.

Figure 2 shows the level values in each dimension of the EQ-5D for each of the eight health states considered. It is based on the average consensus values from the three focus groups rather than the average individual responses before group deliberation. Nominal group discussions involve more reflection and thinking, which provides more considered views than the individuals' first responses [32]. There was a tendency for group deliberation to lead to some health dimensions being judged to be less severe particularly for the most advanced clinical stages.

**Figure 3:**
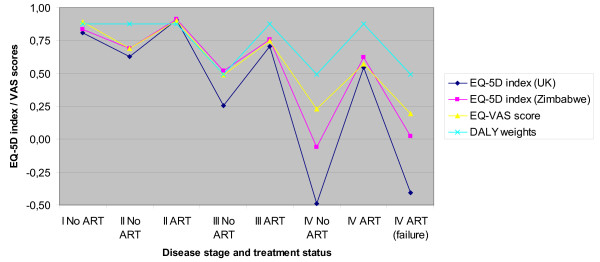
**Average visual analogue scale (VAS) scores and EQ-5D weights (UK and Zimbabwe tariffs) based on expert views**. Disability weights (DALY weights) based on WHO publication.

The associated EQ-5D indices as well as the VAS scores are presented in Figure 3. Please note that the EQ-5D indexes and VAS scores are not strictly comparable without rescaling, since "worst imaginable health" for the latter is anchored at zero while the former allows negative values. The figure illustrates in cardinal terms how quality of life worsens with disease progression. The figure also shows that the improvement in HRQL after introduction of ART is good, but that this effect can be expected to wear off dramatically with the development of drug resistance and treatment failure. For reasons of comparison, the inverted DALY weights from the Global Burden of Disease study [26] have also been included in this figure, using the assumption of Hogan and colleagues that quality of life for people with AIDS receiving ART equals quality of life for people with HIV who have not yet developed AIDS [25]. Note that the clinical experts in this study consider HIV/AIDS to be far more severe than the DALY weights suggest, in particular so for the most advanced disease stages. This tendency was less articulated with the VAS scores than for the indices based on health state descriptions.

It may be noted that even for clinical stage I, the HRQL weight is 0.8, which may not appear intuitive given the fact that this is non-symptomatic HIV. The major reason for this finding is that we stressed that the HIV-status was known to the patients, and the clinical experts therefore judged anxiety and depression to be a problem. For the most severe health states, clinical stage IV without treatment or with treatment failure, the expert opinions resulted in worse-than-death levels for the EQ-5D index when using the UK tariff.

### Discussion

The expert panel in this pilot study reported much lower quality of life weights for advanced and severe AIDS than has typically been found in studies in high income countries [9,33]. A possible reason for this might be better management of opportunistic infection, better nutritional status and general care for AIDS patients in western settings, but it may also be that patients themselves would value their own quality of life more highly than experts do. The first reason seems very plausible, but the fact that the weights for stage III and IV patients in the two South African studies [10,18] are higher than our expert opinions suggest that the question of perspective needs further investigation. In the next phase of this study, we will therefore compare the experts assessment of HRQL presented in this study with those of the patients at the same facilities.

Amartya Sen has suggested that people growing up in a community with much disease burden and few health facilities may be inclined to perceive certain symptoms as more "normal" than people living in well developed communities would do. Hence, he warns that using patients' own perception to evaluate states can be "extremely misleading" [34]. It is therefore not an obvious conclusion that patient preferences are normatively more valid than expert opinion as input into economic evaluation. Rather, we feel that these issues warrant more empirical investigation and debate.

### Conclusions

Our findings suggest that EQ-5D is a good candidate tool for eliciting health related quality of life weights for HIV/AIDS, since both the VAS and the descriptive parts of the tool were relatively easily assessed, produced similar results and were sensitive to differences in health states and availability of treatment. The results suggest that quality of life, as perceived by clinical experts, is strongly correlated to disease stage. Experts also indicate that ART has a strong positive impact on patients' HRQL, although it seems that this treatment effect rebounds dramatically with the occurrence of drug resistance. More research into these areas in clinical settings in sub-Saharan Africa is needed to qualify future economic evaluations on HIV/AIDS treatment and prevention interventions.

## Competing interests

The authors declare that they have no competing interests.

## Authors' contributions

Both authors made equal contributions to conception and design, acquisition, analysis and interpretation of data. Both were involved in drafting the manuscript and both gave final approval of the version to be published.
